# Rab-dependent vesicular traffic affects female gametophyte development in Arabidopsis

**DOI:** 10.1093/jxb/eraa430

**Published:** 2020-09-16

**Authors:** Joanna Rojek, Matthew R Tucker, Sara C Pinto, Michał Rychłowski, Małgorzata Lichocka, Hana Soukupova, Julita Nowakowska, Jerzy Bohdanowicz, Gabriela Surmacz, Małgorzata Gutkowska

**Affiliations:** 1 Faculty of Biology, University of Gdansk, Wita Stwosza 59, Gdansk, Poland; 2 Waite Research Institute, School of Agriculture, Food and Wine, The University of Adelaide, Urrbrae, South Australia, Australia; 3 LAQV REQUIMTE, Departamento de Biologia, Faculdade de Ciências, Universidade do Porto, rua do Campo Alegre s/n Porto, Portugal; 4 Intercollegiate Faculty of Biotechnology, University of Gdansk, Abrahama 58, Gdansk, Poland; 5 Institute of Biochemistry and Biophysics, Polish Academy of Sciences, Pawinskiego 5a, Warsaw, Poland; 6 Institute of Experimental Botany, Czech Academy of Sciences, Rozvojova 263, Praha 6 Lysolaje, Czech Republic; 7 Faculty of Biology, University of Warsaw, Miecznikowa 1, Warsaw, Poland; 8 Ohio State University, USA

**Keywords:** Arabidopsis, auxin transport, female gametophyte, funiculus, ovule, PIN1, PIN3, Rab, rab geranylgeranyl transferase

## Abstract

Eukaryotic cells rely on the accuracy and efficiency of vesicular traffic. In plants, disturbances in vesicular trafficking are well studied in quickly dividing root meristem cells or polar growing root hairs and pollen tubes. The development of the female gametophyte, a unique haploid reproductive structure located in the ovule, has received far less attention in studies of vesicular transport. Key molecules providing the specificity of vesicle formation and its subsequent recognition and fusion with the acceptor membrane are Rab proteins. Rabs are anchored to membranes by covalently linked geranylgeranyl group(s) that are added by the Rab geranylgeranyl transferase (RGT) enzyme. Here we show that Arabidopsis plants carrying mutations in the gene encoding the β-subunit of RGT (*rgtb1*) exhibit severely disrupted female gametogenesis and this effect is of sporophytic origin. Mutations in *rgtb1* lead to internalization of the PIN1 and PIN3 proteins from the basal membranes to vesicles in provascular cells of the funiculus. Decreased transport of auxin out of the ovule is accompanied by auxin accumulation in tissue surrounding the growing gametophyte. In addition, female gametophyte development arrests at the uni- or binuclear stage in a significant portion of the *rgtb1* ovules. These observations suggest that communication between the sporophyte and the developing female gametophyte relies on Rab-dependent vesicular traffic of the PIN1 and PIN3 transporters and auxin efflux out of the ovule.

## Teaser

Defects in Rab prenylation in the *rgtb1* mutant influence the transport of auxin from ovules to the maternal plant and lead to developmental arrest of the female gametophyte in Arabidopsis.

## Introduction

Rab proteins are key components of the vesicular traffic machinery found in all eukaryotes. They reside on the cytosol-facing leaflet of lipid bilayers of organellar membranes. Their interaction with effector proteins enables the recognition and loading of cargo into membrane vesicles, vesicle budding from donor membranes, their movement on the cytoskeleton, and finally recognition, docking, and fusion with acceptor compartment membranes (Pfeffer, 2017). In *Arabidopsis thaliana*, there are 55 Rab genes (Rutherford and Moore, 2002; Shi *et al.*, 2016). Much effort has been directed towards understanding the *in vivo* significance of the GTP/GDP cycle of Rabs (Novick, 2016; Pfeffer, 2017). Less studied is yet another level of Rab activity regulation by lipid modifications on the C-terminal tail. Rabs undergo post-translational modification with two geranylgeranyl moieties on cysteine residues close to the protein C-terminus. This modification enables stable anchoring of Rabs to the membranes, which results in a 10 times higher affinity for the membranes (Silvius and l’Heureux, 1994; Shahinian and Silvius, 1995). The enzyme catalyzing the prenylation of Rab proteins is rab geranylgeranyl transferase (RGT), a complex of catalytic RGTA and lipid substrate-binding RGTB subunits and the accesory Rab escort protein (REP) (Seabra *et al.*, 1992; Thoma *et al.*, 2001). Single geranylgeranylated or unmodified Rab proteins are mistargeted and non-functional (Gomes *et al.*, 2003).

In Arabidopsis, RGTA is encoded by two genes—one probably coding for a non-functional protein—REP is encoded by a single gene, and RGTB is encoded by two functional genes, *RGTB1* and *RGTB2* (Hala *et al.*, 2010; Shi *et al.*, 2016). Total depletion of Rab prenylation by disruption of both *RGTB* genes is lethal, causing pollen sterility (Gutkowska *et al.*, 2015). Disruption of the *RGTB1* gene alone is non-lethal; however, the plants are severely affected (Hala *et al.*, 2010) in a way that can be interpreted as the result of defective auxin gradient formation in the organs; however, the role of auxin in *rgtb1* plants has not been reported.

Auxin, the major plant growth hormone, is synthesized in the shoot apex and in leaf primordia, and is transported to other organs via vascular tissues or cell–cell polar transport (Paque and Weijers, 2016; Mroue *et al.*, 2018). The latter is performed by a set of auxin efflux and influx facilitators, including the PIN and AUX/LAX proteins. PINs are the most important auxin efflux transporters. Their polar localization affects the direction of auxin movement in plant tissues and the formation of auxin gradients during organ growth and differentiation (Petrasek and Friml, 2009; Luo and Zhou, 2018). Polar, asymmetric distribution of PINs on the plasma membrane is regulated by their constant recycling to the endosomes and back by the intracellular vesicle transport machinery (Tanaka *et al.*, 2013; Adamowski and Friml, 2015). The transport of PINs from the *trans*-Golgi network (TGN) to the plasma membrane depends on the activity of small GTPases from the ADP-ribosylation factor (ARF) and RabA families and their regulators (ARF-GEFs and Rab-GEFs) (Geldner *et al.*, 2003; Feraru *et al.*, 2012; Tanaka *et al.*, 2014).

Auxin-dependent processes play an essential role in plant reproduction (Pagnussat *et al.*, 2009; Ceccato *et al.*, 2013; Lituiev *et al.*, 2013; Panoli *et al.*, 2015, Shirley *et al.*, 2019) and the ovule’s response to fertilization (Figueiredo *et al.*, 2015, 2016; Larsson *et al.*, 2017; Robert *et al.*, 2018). A well-coordinated spatiotemporal network of auxin production, transport, and signaling is critical for synchronized development of the sporophytic part of the ovule and female gametophyte (FG; Robert *et al.*, 2015).

The plant ovule is a fundamental organ for reproduction, and is the site of megaspore formation (megasporogenesis), FG formation (megagametogenesis), fertilization, and embryo and endosperm development (Shirley *et al.*, 2019). In Arabidopsis, ~50 ovules are formed inside the pistil of each flower. During female germline development, a single archesporial cell differentiates and undergoes meiosis in each ovule (Pinto *et al.*, 2019). Out of the four post-meiotic spores, only one survives, and develops to form a seven-celled FG, also called the embryo sac (Webb and Gunning, 1990; Christensen *et al.*, 1997). Concurrently, the surrounding sporophytic tissues grow to form integuments (Schneitz *et al.*, 1995). The ovule remains connected with the maternal plant via the funiculus—a stalk filled with vascular tissue. The funiculus enables direct transport of hormones and nutrients to and from the developing ovule (Khan *et al.*, 2015). Two sperm cells delivered by a pollen tube fertilize the central cell and the egg cell. After double fertilization (Zhou and Dresselhaus, 2019), the zygote develops into the embryo, while the fertilized central cell develops into a nutritional tissue, the endosperm (Brown *et al.*, 1999; Faure *et al.*, 2002).

In this study, we aimed to address the role of vesicular transport in mediating interactions between sporophytic and gametophytic tissues within the ovule: specifically, does vesicular transport deficiency in the sporophyte influence the fate of the developing FG and what transport-related mechanisms are involved? To this end, we chose to investigate the *rgtb1* mutant which, unlike many other transport-related mutants, is not embryo-lethal. Rather, the *rgtb1* mutant produces viable sporophytes, in addition to defective FGs at reasonably high frequency.

Our findings show that interfering with vesicle traffic affects PIN1 and to a lesser extent PIN3 recycling from the endosomes to the basal membranes of the funiculus, and prevents auxin efflux from the ovule. In particular, PIN1 internalization during meiosis/early FG development leads to increased auxin concentration in the ovule and the arrest of FG development at the functional megaspore (FM)/FG2 stage. In some *rgtb1* ovules, the presence of RGTB2 activity is apparently sufficient to rescue the RGTB1 deficiency. This highlights the importance of RGTB1-mediated vesicular transport in sporophytic tissues during ovule development and FG formation.

## Materials and methods

### Plant material

Plant lines used were: Arabidopsis wild type (WT) Col-0, *rgtb1-1* (SALK 015871), and *rgtb1-2* (SALK 125416) as in Hala *et al.* (2010) and Gutkowska *et al.* (2015). PIN1–green fluorescent protein (GFP), was from the Nottingham Arabidopsis Stock Centre (NASC), number N9362 (Benkova *et al.*, 2003), PIN3–GFP (Zadnikova *et al.*, 2010), was a gift from Dr Katerina Schwarzerova (UEB CAS, Prague), DII-Venus, was NASC number N799173 (Brunoud *et al.*, 2012), and *pDR5rev:*3×Venus, was NASC number N799364 (Heisler *et al.*, 2005). *rgtb1-1* and *rgtb1-2* heterozygous plants were crossed to fluorescent marker lines, and homozygous plants were identified in the F_2_ generation by phenotyping and genotyping with appropriate primer pairs. Due to low fertility, the *rgtb1* lines were maintained as segregating populations. F_2_ and further generations were used for microscopic analysis. For reciprocal inheritance crosses, heterozygous *rgtb1* plants were chosen by PCR genotyping. The progeny of each cross was grown in soil for 1 month and PCR genotyped; 170–450 plants of each cross were analyzed.

For flower and ovule observations, plants were grown in soil in long-day conditions (16 h light, 8 h darkness). Homozygous plants were chosen by means of their characteristic dwarf phenotype.

### Scanning electron microscopy

For SEM observations, flowers were fixed in 3% glutaraldehyde in 25 mM phosphate buffer (pH 7.2) overnight, rinsed, dehydrated and critical-point dried, coated with a thin gold layer, and examined using a LEO 1430VP scanning electron microscope (Carl Zeiss, Germany).

### Sample clearing

Flower buds were fixed in acetic acid:ethanol 1:3 solution and cleared in chloral hydrate solution [66.7% chloral hydrate (w/w), 8.3% glycerol (w/w)] or cedar oil as described (Rojek *et al.*, 2018). Ovules were examined under a Nikon Eclipse E800 epifluorescence microscope equipped with differential interference contrast (DIC) optics and a Nikon DS-5Mc CCD camera (PRECOPTIC Co.).

### Fluorescence analysis of ovules

For fluorescence analysis, the ovules were mounted in 7% glucose. For FM^®^ 4-64 dye application (Invitrogen), dissected ovules were incubated in 4 µM FM4-64 diluted in 7% glucose, on glass slides for 1–2 h in the dark and observed using confocal laser scanning microscopy (CLSM; Leica TCS SP8X). To avoid differences in fluorescence intensity due to transcript silencing in consecutive generations, sister (progeny of the same mother plant) WT and *rgtb1* plants were used for imaging. Specimens were imaged using an epifluorescence microscope (Nikon Eclipse E800) or CLSM. Detection of GFP and yellow fluorescent protein (YFP; Venus) under the epifluorescence microscope was achieved with Epi-Fl Filter Block B-1E (EX 470–490, DM 505, BA 520–560). A filter Block G-2A (EX 510–560, DM 575, BA 590) was used for co-localization of non-specific fluorescent signal and cuticule-like components. For the sake of image clarity, a uniform procedure was applied: ovules at stages younger than FG4 were imaged at ×100 magnification and older ovules starting from FG4 were imaged at ×40 magnification. Roots in control experiments were obtained from 7-to 10-day-old seedlings grown vertically on 1/2 Murashige and Skoog (MS) medium without sugar, and 1.5% agar.

Under CLSM, the GFP excitation wavelength was set to 489 nm and emission was detected at 505–547 nm; for YFP (Venus), excitation was at 514 nm and emission was at 524–566 nm; and for FM4-64, excitation was at 558 nm and emission was at 674–766 nm. CLSM imaging was performed with a ×63 oil immersion objective.

All figures were prepared in Adobe Photoshop (Elements 11 and CS6 versions).

### Statistical analysis

All calculations were performed in GraphPad Prism 5.0 software. Mean values and standard error of the mean were calculated and data were compared with unpaired Student *t*-test with a two-tailed hypothesis. Graphs were prepared using the same software. In every case, several (at least three) independent plant cultivations, each of at least 10 plants per genotype, were performed to generate data. A Fisher exact test against the H_0_ hypothesis that the allele transmissions are equal was applied in the case of reciprocal genetic cross analysis.

### Transcriptomic analysis

RNASEQ reads from the experiment SRP075604 were downloaded from publicly available databases (Klepikova *et al.*, 2015, 2016). Reads were mapped to the Arabidopsis genome and gene expression was calculated [in transcripts per million (TPM)] using CLC Genomics workbench ver9.5.2 (www.clcbio.com). For the purpose of this study, only selected stages and tissues were analyzed in detail. Normalized gene expression values for the Col WT nucellus, FG, and whole ovule were extracted from microarray data reported previously (Tucker *et al.*, 2012a). Presence/absence tags were used to assess whether genes were expressed; values below an arbitrary expression value of 10 generally indicate that the transcript is undetectable.

### mRNA *in situ* hybridization

Inflorescences from Col-0 WT plants were fixed in FAA (50% ethanol, 5% acetic acid, 4% paraformaldehyde, and 0.025% Tween-20) and embedded in paraffin as described previously (Tucker *et al.*, 2003). To generate the probe, an *RGTB1* fragment was amplified from genomic DNA using the following oligonucleotides with T7 adaptors: RGTB1_ASF (5'-TGGTCAAACAATATGGCCG), RGTB1_ASR (5'-TAATACGACTCACTATAGAGCAGCAACACAACTTCGTT), RGTB1_SF (AGCAGCAACACAACTTCGTT), and RGTB1_SR (TAATACGACTCACTATAGTGGTCAAACAATATGGCCG). Digoxigenin (DIG)-labeled probes were transcribed with T7 polymerase using the DIG-labeling kit (Roche). *In situ* hybridization was performed using an InsituPro VSi robot (Intavis), following a standard protocol (Javelle *et al.*, 2011).

## Results

### *rgtb1* produces deformed flowers

**Fig. 1.**
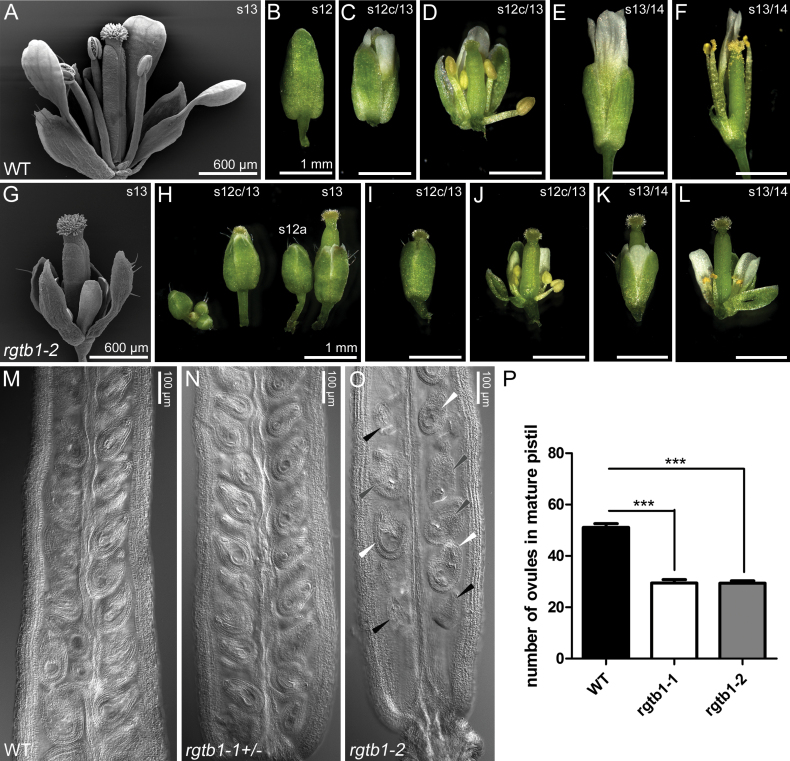
The *rgtb1* mutation disturbs flower development and reduces fertility. Selected developmental stages of WT (Col-0, A–F) and *rgtb1-2* (G–L) plants. Delay of stamen and vegetative organ growth in comparison with the pistil (G), precocious bud opening, and loss of typical anthesis at stage 13 (H–K). Problems with pollen release (L). (M and N) Ovules in WT (M) and *rgtb1*+/– (N) flowers around anthesis. (O) Empty ovules of normal size (black arrowheads) or collapsed ovules (white arrowheads) accompany normal mature ovules in *rgtb1*–/– flowers around anthesis. (P) Number of ovules in mature pistils (from flowers around anthesis). Number of pistils counted: WT *n*=25, *rgtb1-1 n*=24, *rgtb1-2 n*=36. Bars represent the mean ±SEM compared with the unpaired Student *t*-test. Results are highly significant (*P*<0.001). (A, G) SEM; (B–F, H–L) stereomicroscopy. Scale bar=1 mm in (B) and (H) which are shown at scale with the photographs in (C–F) and (I–L). Photograph of a cleared pistil under DIC on a microscope (M–O). (This figure is available in color at *JXB* online.)

The general features of *rgtb1-1* and *rgtb1-2* plants were described previously (Hala *et al.*, 2010), with both alleles showing similar phenotypes. *rgtb1* flowers are smaller than those of the WT, and the perianths never open (Fig. 1A–F versus G–L). In mature *rgtb1* flowers, a relatively long pistil is surrounded by a normal number of small sepals, petals, and anthers (Fig. 1G). The flower phenotypes of *rgtb1* and the lack of typical anthesis make it difficult to establish the actual stage of flower and gametophyte development. To overcome this, we present the comparison of WT versus *rgtb1* flowers based on pistil and stigma maturity according to Smyth *et al.* (1990) and Christensen *et al.* (1997) which we use throughout this work (Fig. 1B–F, H–L).

The discrepancy between pistil and anther size was proposed to account for the low fertility of *rgtb1* mutants (Hala *et al.*, 2010) together with pollen coat defects and pollen germination deficiency (Gutkowska *et al.*, 2015). However, closer inspection of *rgtb1–/–* flowers revealed a lower number of ovules per mature ovary than in the WT or *rgtb1+/–*, and many of the ovules, despite normal size, were deformed (Fig. 1M–P). The observations of ovule deformation were new in the context of *rgtb1* mutations, hence we decided to study the reason for the reduced fertility on the female side.

### *rgtb1* ovule defects are sporophytic in origin

The fact that *rgtb1* homozygous mutants are viable indicates that any gametophytic effect of *RGTB1* mutation is not completely penetrant. On the other hand, *rgtb1–/–* plants are nearly infertile. In order to provide evidence that infertility in *rgtb1* is of sporophytic origin, we compared the transmission efficiency through the male and female gametes in heterozygous *rgtb1* plants by backcrossing them to the WT, as both pollen donors and acceptors (Table 1). Although transmission through the male gamete was decreased to ~60–70% in *rgtb1* mutants, no significant change in transmission efficiency through the female gamete was observed. This result suggests that any defects in FG development and/or fertilization in *rgtb1* mutants are dependent on RGT activity in sporophytic tissues. Consistent with this, in *rgtb1–/–* mother plants, we observed deformed and non-viable ovules, while in *rgtb1+/–* mother plants, apparently normal ovules containing a *rgtb1*– FG were functional and capable of normal genetic transmission of the allele to the progeny.

**Table 1.** The *rgtb1* is a recessive sporophytic mutation

Pollen acceptor/pollen donor	Expected *rgtb1 +/–*:WT ratio	Obtained *rgtb1 +/–*:WT ratio	Transmission of the *rgtb1* allele	*P*-value (Fisher exact test)
WT×*rgtb1*-*1+/–*	1:1	58:114	0.34	0.0031**
*rgtb1*-*1+/–*×WT	1:1	107:130	0.45	NS
WT× *rgtb1*-2*+/–*	1:1	150:270	0.36	<0.0001***
*rgtb1*-2*+/–*×WT	1:1	229:218	0.51	NS

### Functional megaspore arrest leads to defective female gametogenesis in the *rgtb1* mutant

We examined female sporogenesis in *rgtb1* homozygous plants in comparison with WT plants. No differences were observed in megaspore mother cell (MMC) differentiation, meiotic division, or FM differentiation (Supplementary Fig. S1 at *JXB* online; >600 ovules were observed for each genotype).

**Fig. 2.**
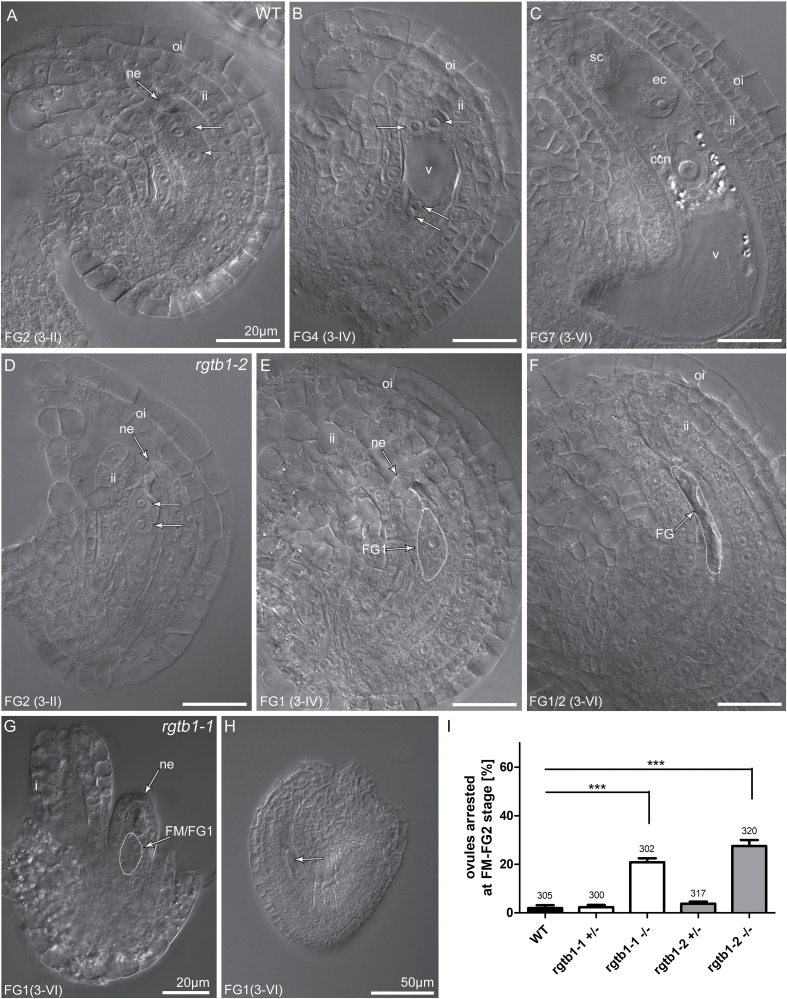
The *rgtb1* mutation affects ovule and female gametophyte (FG) development. Stages of ovule development in WT (A–C), *rgtb1*-*1* (G and H), and *rgtb1*-*2* (D–F) ovules from flowers at developmental stage 3-I to 3-VI (according to Schneitz *et al.*, 1995; Christensen *et al.*, 1997). (A–C) WT plants; stages of FG development correlate with ovule development. Starting from the 3-I stage (corresponding to the FG2 stage of the ovule in the WT), a developmental arrest is observed in a large portion of *rgtb1* ovules at the FM or FG1 stage. Normal development of ovules at the 3-II/FG2 flower stage (A versus D), developmental arrest of the FG in *rgtb1* at the 3-IV/FG4 flower stage (B versus E), and the 3-VI/FG6 flower stage (C versus F–H) with normal (E, F, H) or (D, G) abnormal development of integuments. (I) Fraction of ovules arrested at the FM/FG2 stage of gametogenesis (%). In each case, >300 ovules were counted, while the exact number is given above the corresponding bar. Bars represent the mean ±SEM. Data were compared with unpaired Student *t*-test; ***indicates a *P*-value <0.001. (A–H) DIC microscopy. Abbreviations: ne, nucellar epidermis; ii, inner integument; oi, outer integument; ec, egg cell; sc, synergid cell; ccn, central cell nucleus; v, central vacuole; FM, functional megaspore; FG, female gametophyte. Nuclei in FGs are marked by arrows (A, B, D). Scale bar=20 μm for all images.

Abnormalities in *rgtb1* ovules were first detected after the FM stage in comparison with the WT (Fig. 2A–C versus D–H; I). Approximately 21–27% of ovules in *rgtb1*-*1* and *rgtb1*-2 homozygous plants, respectively, arrested at this stage (Fig. 2E, G, H) or immediately after the first mitotic division—at the bi-nucleate stage (Fig. 2F)—in comparison with only 2–4% of ovules in WT or heterozygous plants (~300 ovules were counted for each genotype). This observation confirmed the result of the reciprocal crosses, which suggested that sporophytic defects are responsible for abnormal ovule development (Table 1). Deformation of the integuments was also observed in *rgtb1*–/– plants (Fig. 2D, G). The inner or outer integument was not present, and instead the FG cells protruded or the integuments grew asymmetrically.

These abnormal phenotypes are reminiscent of ovules showing deficiencies in auxin biosynthesis and flux (Schruff *et al.*, 2006). For example, hypomorphic *pin1-5* mutants show similar defects in the ovule to those observed in *rgtb1* (Ceccato *et al.*, 2013). Because auxin transport by PIN proteins is Rab vesicle dependent, and PIN1 and PIN3 are the main auxin efflux proteins in the developing ovule, we decided to study PIN1 and PIN3 protein localization in *rgtb1* ovules.

### PIN1–GFP and PIN3–GFP are internalized from basal membranes in the funiculus provascular cells of *rgtb1* ovules

We crossed *rgtb1* plants to the *pPIN1:*PIN1-GFP (Benkova *et al.*, 2003) and *pPIN3*:PIN3-GFP (Zadnikova *et al.*, 2010) lines, markers for auxin efflux in multiple organs including the ovule, and analyzed homozygous progeny of the cross. Localization of PIN1–GFP in root tissues of the seedlings was indistinguishable between WT and *rgtb1* plants (Supplementary Fig. S2A, B versus C–F), while PIN3–GFP marked fewer cells in the quiescent center (QC) of the root meristem of *rgtb1*-*1* than in the WT (Supplementary Fig. S2G–I versus J–L).

**Fig. 3.**
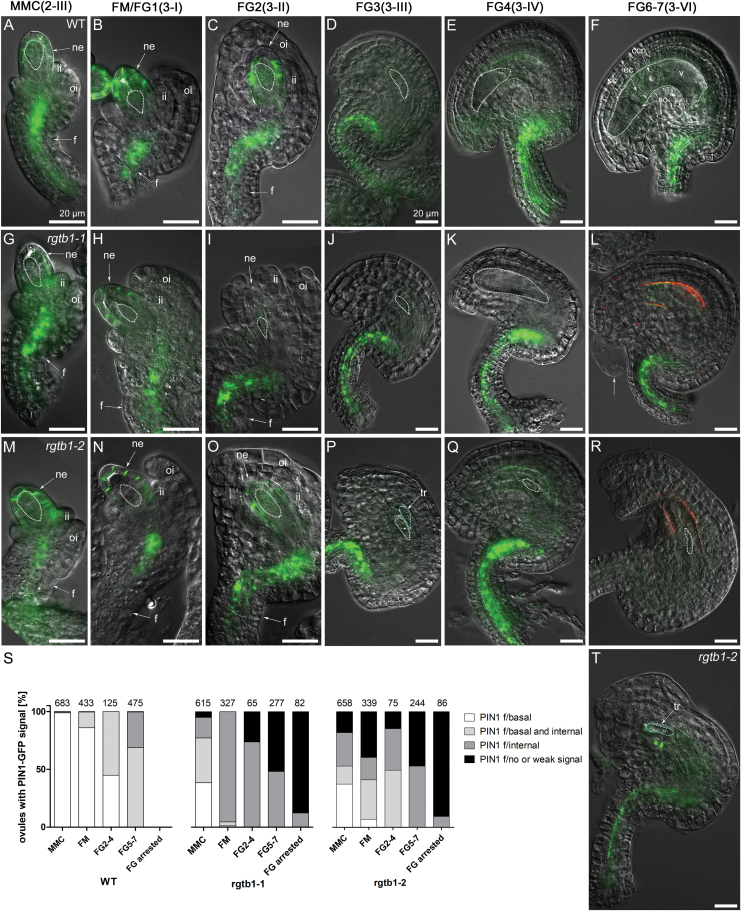
PIN1 is localized on polar membranes at the tip of the *rgtb1* ovule nucellus, but mislocalized in provascular cells of the funiculus. *pPIN1:*PIN1*-*GFP expression in WT (A–F), *rgtb1-1* (G–L), and *rgtb1-2* (M–R, T) ovules from stages 2-III to 3-VI (according to Schneitz *et al.*, 1995). During stages 2-III/MMC to 3-II/FG2, PIN1–GFP is polarly localized at the tip of the nucellus in WT and *rgtb1* ovules (A–C versus G–I, M–O). Decreased PIN1 expression in the nucellus starts from stage 3-III/FG3, leading to an absence of nucellar signal in all ovules at later stages (D–F versus J–L, P–R). Basal polar signal in the WT funiculus provasculature at the MMC stage becomes gradually internalized at ovule maturity (examples of basal polar signal A–C and partial internalization D–F). (S) Quantification of ovules expressing PIN1–GFP according to the genotype and developmental stage. Nucellar signal in WT and *rgtb1* ovules was uniform; therefore, only the differences of funiculi-localized signal were considered. The range of phenotypes of PIN1–GFP localization in *rgtb1* with funiculus provasculature signal were divided into four classes: basal polar localized (white, examples in M, N); partially internalized (light gray, examples in L, Q); intracellular (dark gray, examples in (G, H–K, O–P); absent or very weak (black, example on R, T). FM/FG2-arrested ovules isolated from older ovaries were considered in a separate class. The number of ovules counted is given above each bar. (T) Example of an *rgtb1-2* ovule developmentally impaired with underdeveloped integuments, lacking the FG and with a tracheary element-like structure adjacent to the aborted FG. (A–R, T) Epifluorescence microscopy; DIC and PIN1–GFP signal. The white dotted line highlights the MMC, FM, or FG, as appropriate for the image. Abbreviations: I, integuments; ii, inner integument; oi, outer integument; ne, nucellar epidermis; f, funiculus, ec, egg cell; sc, synergid cell; ccn, central cell nucleus; v, central vacuole; tr, aborted tracheary element; mmc, megaspore mother cell; FM, functional megaspore; FG, female gametophyte. Scale bar=20 μm on all images. (This figure is available in color at *JXB* online.)

Using fluorescence microscopy in combination with DIC, we precisely revealed the tissue- and stage-specific altered expression of PIN1–GFP in *rgtb1* ovules. In WT ovules containing an MMC and at the conclusion of megasporogenesis, PIN1–GFP showed polar localization in the most distal nucellar epidermal cells. This asymmetric membrane localization of PIN1–GFP was comparable between the WT and *rgtb1* (Fig. 3A, B versus G, H, M, N). PIN1–GFP expression gradually decreased in the WT and *rgtb1* nucellus, starting from the first mitotic division of the FG, and it was undetectable in ovules collected from mature WT and *rgtb1* flowers (Fig. 3C–F versus I–L, O–T); at that stage, PIN1–GFP was restricted to the chalaza (a medial domain connecting the funiculus to the rest of the ovule) and the funiculus.

**Fig. 4.**
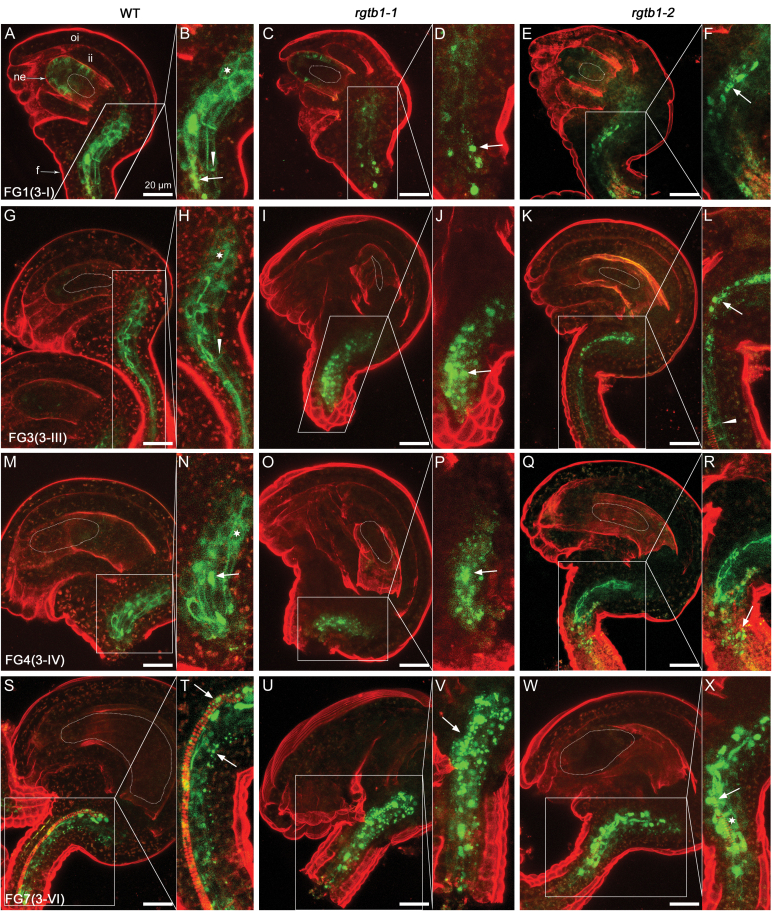
PIN1–GFP is internalized in the funiculus provasculature of *rgtb1* ovules. *pPIN1*:PIN1–GFP expression in WT (A, B, G, H, M, N, S, T), *rgtb1*-*1* (C, D, I, J, O, P, U, V), and *rgtb1*-*2* (E, F, K, L,Q, R, W, X) ovules from developmental stage 3-I to 3-VI (according to Schneitz *et al.*, 1995). (A and B) PIN1–GFP is basal polarly localized in provascular cells of the funiculus in the young WT ovules (at the FG1 stage) and with progression of development becomes partly internalized (G and H, at the FG3 stage; M and N, at the FG4 stage) and is internalized in mature ovules (S and T). In a large fraction of *rgtb1* ovules, the PIN1–GFP signal is internalized, at least partially, throughout the whole of development, starting from the unicellular stage until mature FG (*rgtb1*-*1* images C, D, I, J, O, P, U, V; *rgtb1*-*2* images E, F, K, L, Q, R, W, X). (A–X) Confocal laser scanning microscopy, PIN1–GFP signal and FM4-64 dye fluorescence. Dotted lines highlight the MMC, FM, or FG, as appropriate for the image. Abbreviations: ne, nucellar epidermis; f, funiculus; ii, inner integument. Basal polar localization of PIN1–GFP is marked by arrowheads, intracellular localization is marked by arrows, and an asterisk marks cells with apolar PIN1–GFP localization. The left narrow image represents a magnification of the area boxed in the right image. Scale bar=20 μm for all images. (This figure is available in color at *JXB* online.)

In funiculi, the localization of PIN1–GFP appeared different between the WT and *rgtb1* mutants. Therefore, we used CSLM in ovules stained with FM4-64 styryl dye to assess PIN1–GFP subcellular localization in that tissue (Fig. 4). Based on images obtained by fluorescence microscopy with DIC imaging (Fig. 3) and CLSM (Fig. 4), we calculated the frequency of ovules presenting PIN1–GFP localized only on the basal plasma membrane of the funiculus cells, partially internalized from the basal polar membrane, only endosomal, and lacking the PIN1–GFP signal. In the WT at the MMC stage, PIN1–GFP showed basal polar localization in all ovules (Fig. 4A, B). During subsequent development, PIN1–GFP signal was gradually internalized, with >50% of ovules at the FG4 stage showing at least partial endosomal localization (Fig. 4M, N). In the WT, all mature ovules had PIN1–GFP signal predominantly internalized (Figs 3S, 4S, T).

In *rgtb1*, similar to what is described in Fig. 2 for ovule development, we observed a range of different PIN1–GFP phenotypes in the funiculus at any developmental stage. In striking contrast to the WT, already before meiosis, PIN1–GFP started to be internalized in 36–50% of *rgtb1* funiculi (Fig. 3G). At the same stage, ~30% of *rgtb1* ovules showed normal WT-like signal and few lost the signal from the funiculi (Fig. 3M). At the FM/FG1 stage, nearly all *rgtb1* ovules had PIN1–GFP signal internalized or even absent, depending on the genotype (Fig. 3S), while at this stage in the WT only 10% of ovules showed evidence of PIN1–GFP internalization. Examples of *rgtb1* ovules with the endosomal signal at the FM/FG1 stage are shown in Fig. 4C–F. At later stages of development, the signal of PIN1–GFP in *rgtb1* funiculi was always internal or absent, and the fractions of ovules that lacked the PIN1–GFP in funiculi increased during development, reaching 15–25% at FG2–FG4 stages and 50% at the FG6/7 stages (Figs 3S, 4I–L, O–R, U–X). In the WT at maturity, the PIN1–GFP signal was also internalized, but was present in all analyzed ovules.

Note that *rgtb1 pPIN1*:PIN1-GFP showed the same frequency of ovules arrested at the FM/FG1/FG2 stage as the mutant *rgtb1*. Sister ovules coming from the same ovary showed either normal or precocious PIN1–GFP internalization and were distributed randomly.

**Fig. 5.**
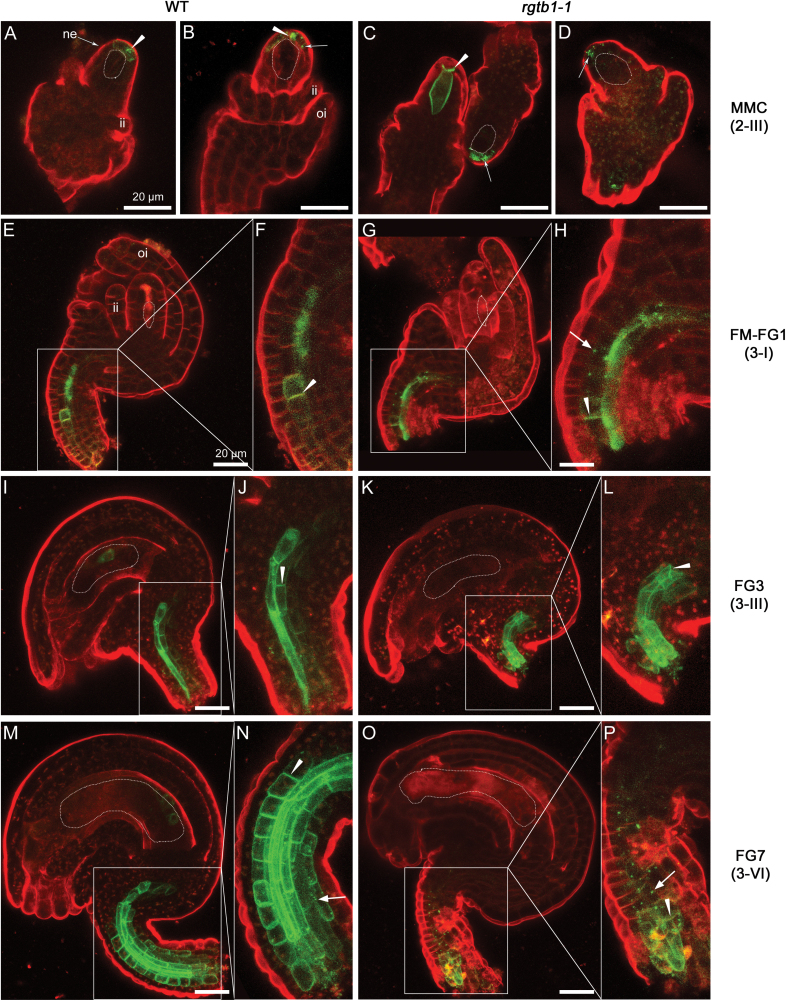
PIN3–GFP is partly internalized in the funiculus provasculature of *rgtb1* ovules. *pPIN3*:PIN3-GFP expression in WT (A, B, E, F, I, J, M, N) and *rgtb1*-*1* (C, D, G, H, K, L, O, P) ovules from developmental stage 2-III to 3-VI (according to Schneitz *et al.*, 1995). PIN3–GFP localizes to MMC-adjacent membranes of the nucellus at the tip of the ovule in the WT and *rgtb1*-*1* (A and B versus C and D); at later stages, the nucellar signal disappears in both genotypes (E–P). PIN3–GFP is also basal–polar localized in provascular cells of the funiculus in WT plants starting from the FM stage (E, F for the FM/FG1 stage; I, J for the FG3 stage; M, N for mature ovules). In *rgtb1*-*1*, PIN3–GFP is expressed in the same regions of the funiculus but is internalized to a larger extent (G, H versus E, F; K, L versus I, J; O, P versus M, N). (A–P) Confocal laser scanning microscopy, PIN1–GFP signal and FM4-64 dye fluorescence. Dotted lines highlight the MMC, FM, or FG, as appropriate for the image. Abbreviations: ne, nucellar epidermis; f, funiculus; ii, inner integument; oi, outer integument. Basal polar localization of PIN3–GFP is marked by arrowheads, and intracellular localization is marked by arrows. The left narrow image represents magnification of the area boxed in the right image. Scale bar=20 μm for all images. (This figure is available in color at *JXB* online.)

Both in WT and in *rgtb1*-*1* ovules, PIN3–GFP signal became detectable in a few cells at the tip of the nucellus (Fig. 5A, B versus C, D), on the membranes that contact directly with the MMC, in agreement with earlier studies (Ceccato *et al.*, 2013). No signal of PIN3–GFP was detected at the chalazal or funiculi at the MMC stage in both genotypes (Fig. 5A–D), in contrast to a well-established signal for PIN1–GFP (Fig. 3A, G, M, S). Before meiosis, WT and *rgtb1*-*1* ovules showed a similar pattern of PIN3–GFP (Fig. 5A, B versus C, D). After meiosis, PIN3–GFP fluorescence disappeared from the micropylar pole of the ovule, and became visible in the funiculus provasculature. In the WT, PIN3–GFP was localized on the plasma membrane, but not strictly in a basal polar manner (Fig. 5E, F), while in *rgtb1*-*1* it was partly internalized in nearly half of the ovules (Fig. 5G, H). This pattern was preserved in later stages of development (Fig. 5I, J for the WT versus K, L for the mutant). Finally, in mature WT ovules, PIN3–GFP localization became polarized in funiculus provascular cells (Fig. 5M, N), similar to earlier reports (Larsson *et al.*, 2017). In all defective FM/FG2-arrested ovules isolated from mature ovaries of *rgtb1*-*1*, the PIN3–GFP signal remained at least partly intracellular (Fig. 5O, P).

### Auxin accumulates in arrested ovules of the *rgtb1* mutant

In order to analyze auxin accumulation in the developing ovule, we used two auxin reporters that rely on different principles of operation. The first was *pDR5rev*:3×Venus-N7 (Heisler *et al.*, 2005). This construct measures the auxin signaling output by inducing the synthesis of a 3×Venus fluorescent protein bearing a nuclear localization signal (NLS). The protein is transcribed from the *Cauliflower mosaic virus* (*CaMV*) *35S* minimal promoter preceded by a regulatory element that binds the ARF protein–auxin complex. Hence the cellular response to active auxin can be measured. The second construct was *35S:*DIIS-Venus-NLS, which gives results complementary to those from the *pDR5rev*:3×Venus construct (Brunoud *et al.*, 2012). DIIS-Venus measures the auxin signaling input. It consists of the coding sequence of the naturally existing Aux/IAA domain, which is quickly degraded by the proteasome upon auxin binding, fused to a nuclear-localized version of the Venus protein. The construct is transcribed from the *CaMV35S* promoter in almost every plant cell; in the presence of auxin, the reporter protein is degraded and no signal is detected. In the case of cells in which auxin is transiently present (is only transported through), the readout from *pDR5rev:*3×Venus-N7 may be (nearly) negative while the readout from DIIS-Venus or the related R2D2 construct is positive (Robert *et al.*, 2015).

In control experiments in both WT and *rgtb1* plants, the *pDR5rev:*3×Venus fluorescent signal was present in the QC of the root apical meristem, in the root epidermis, and in vascular strands (Supplementary Fig. S2M–O), while the DIIS-Venus reporter fluorescence was present only in cell nuclei of elongated epidermal cells in the root hair formation zone (Supplementary Fig. S2P–R).

**Fig. 6.**
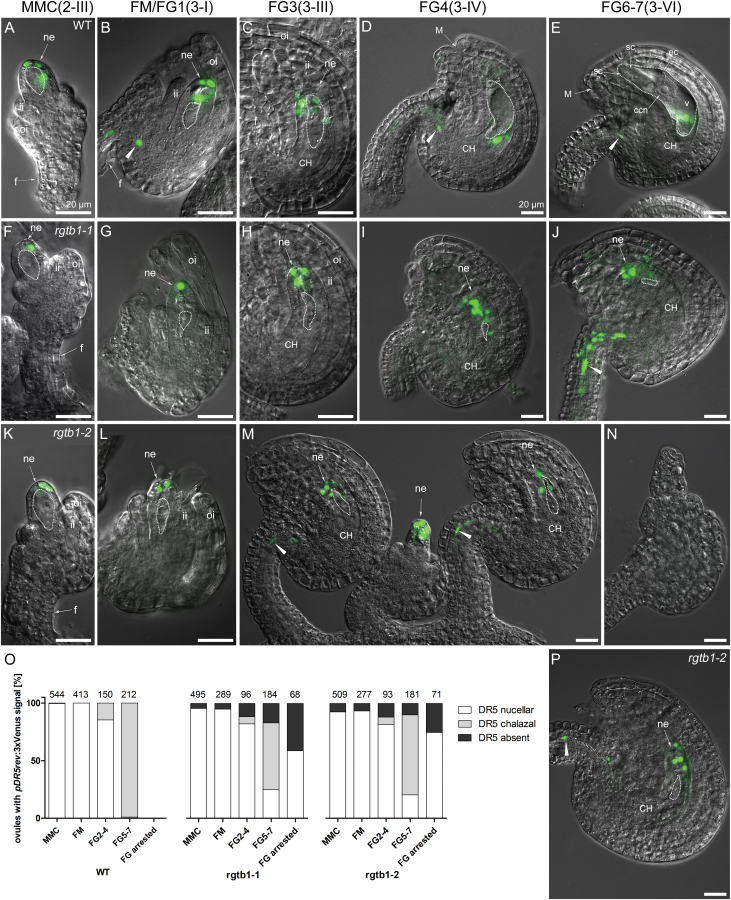
Developmentally impaired *rgtb1* ovules show mislocalized response of the *DR5rev:*3×Venus auxin reporter. Auxin nuclear reporter *DR5rev:*3×Venus expression in WT (A–E), *rgtb1-1* (F–J), and *rgtb1-2* (K–N, P) ovules from developmental stage 2-II to 3-VI (according to Schneitz *et al.*, 1995). *DR5rev:*3×Venus signal at the tip of the nucellus of WT and *rgtb1* ovules at the 2-II/MMC stage (A versus F, K) until stage 3-III/FG3 (B, C versus G, H, L, M). In WT ovules from stage 3-IV/FG4 until maturity, *DR5rev*:3×Venus signal is localized at the chalazal part of the ovule (D, E; DR5 signal from the chalazal nucellus layer overlaps the FG layer) and the funiculus provasculature. In many *rgtb1* cases, *DR5rev*:3×Venus signal remains localized in the micropylar pole of the ovule (for stage FG4, D versus I, M; for stage FG6, E versus J, P) and usually is also present in the funiculus. (M) *rgtb1*-*2* ovules showing normal *DR5rev*:3×Venus signal localization at stage 3-III/FG3 and, in the middle, a developmentally arrested ovule expressing *DR5rev*:3×Venus at the tip of the nucellus. (N) Ovule arrested at an early stage of development showing no signal of *DR5rev*:3×Venus. (O) Fractions of ovules at different developmental stages from plants showing *DR5rev*:3×Venus reporter activity. Ovules were divided into three classes based on the presence of *DR5rev:*3×Venus signal at the top of the micropylar nucellus (white, examples in A–C, F––J, K–M, P), at the chalazal pole of the ovule in the endothelium (gray, examples in D, E), or with the signal absent (black, example in N). Ovules were counted under fluorescent and confocal microscopes. The number of ovules counted is given above each bar. (A–N, P) Epifluorescence microscopy; merged images of DIC and the *DR5rev:*3×Venus signal. The white dotted line highlights the MMC, FM, or FG, as appropriate for the image. Abbreviations: ii, inner integument; oi, outer integument; f, funiculus; ne, nucellar epidermis; CH, chalaza, M, micropyle; ec, egg cell; sc, synergid cell; ccn, central cell nucleus; v, central vacuole. Arrowheads point to the *DR5rev*:3×Venus signal in the funiculus. Scale bar=20 μm for all images. (This figure is available in color at *JXB* online.)

Again, we decided to use DIC contrast imaging on whole-mount ovules to clearly distinguish stages of development of the FG. During early ovule development, the activity of the auxin reporter *pDR5rev*:3×Venus was similar in both WT and *rgtb1* plants. From the MMC stage until the FG3 stage, signal was detected in the most distal epidermal nucellar cells (Fig. 6A–C versus F–H, K–M). While the *pDR5rev*:3×Venus signal mirrors the PIN1–GFP- and PIN3–GFP-expressing cells at the tip of the nucellus, the high expression of PIN proteins at the chalaza and funiculus is surprisingly not accompanied by the auxin reporter *pDR5rev*:3×Venus. This was similar in WT and *rgtb1* plants at early stages of FG development, and is consistent with previous observations (Bencivenga *et al.*, 2012; reviewed in Robert *et al.*, 2015).

From the FG4 stage onwards, the nucellar tissue surrounding the FG continued to degenerate and cells with reporter activity were found in the chalazal pole, but always outside the FG in WT ovules, even at maturity (Fig. 6D, E). Generally, throughout FG development, the *pDR5rev*:3×Venus signal decreased, but persisted in the funiculus. At the time of second mitosis in the FG, 16% of WT ovules showed an auxin response at the chalazal end of the ovule (Fig. 6D) whilst a similar fraction of *rgtb1* ovules completely lacked signal. During later stages of development, 21–25% of *rgtb1* ovules were arrested at FM/FG2 and showed *pDR5rev:*3×Venus activity in the epidermal nucellar cells, a feature characteristic of earlier stages of development (Fig. 6D, E versus J, P). Conversely, *pDR5rev:*3×Venus activity was not detected at all in the most severely affected *rgtb1* ovules at any of the analyzed stages (Fig. 6N, 5–17%). Still, >50% of the ovules isolated from mature *rgtb1* ovaries expressed *DR5rev*:3×Venus in a WT-like manner, namely at the chalazal pole and in the funiculus (Fig. 6O). As was the case for PIN1–GFP, ovules arrested at the FM/FG2 stage with the micropylar auxin maximum and ovules progressing normally through development were randomly distributed in each ovary (Fig. 6M).

**Fig. 7.**
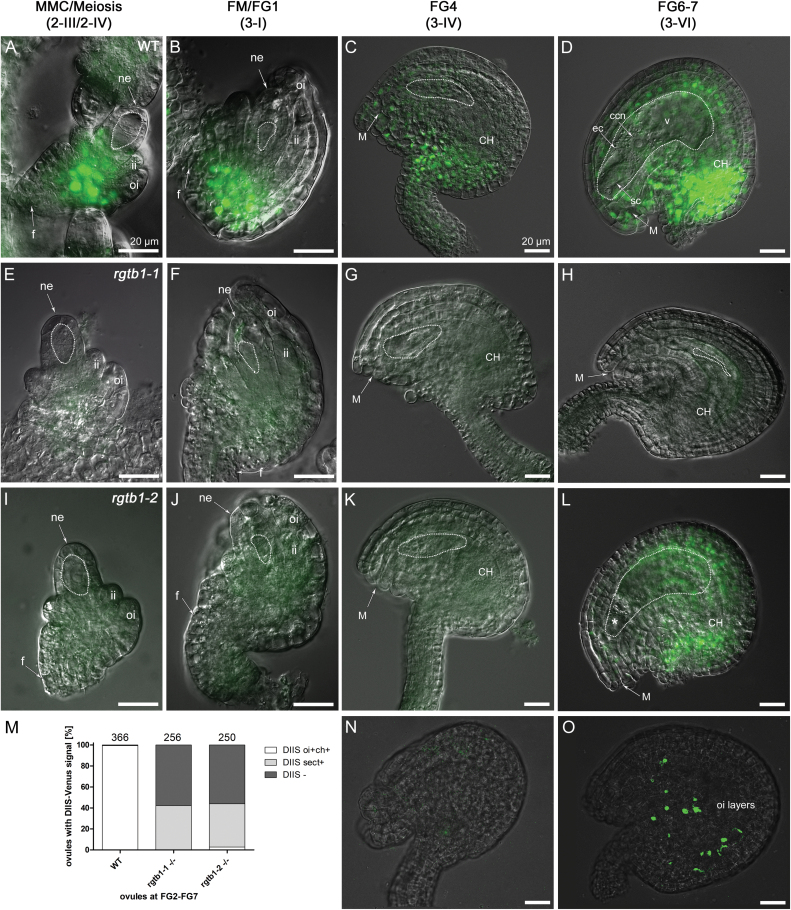
The *35S:*DIIS-Venus auxin sensor signal is decreased in *rgtb1* ovules. The *CaMV35S:*DIIS-Venus signal during ovule development in WT (A–D) and *rgtb1* (E–L, N, O) ovules. (A) Meiosis, WT; the DIIS-Venus signal is in the chalaza. At FM and early FG stages, the DIIS-Venus fluorescence is also present in the outer integument (B, C). Around maturity, DIIS-Venus is detected in the integuments, the chalazal pole, and in the outer cell files of the funiculus (D). At no stage was DIIS-Venus fluorescence seen within the WT FG (D). DIIS-Venus signal was lacking in all young *rgtb1* ovules (E, F, I, J) and in 60% of ovules around maturity, both with an arrested (H, N) and with a normal FG (G, K, L). At maturity, <3% of the *rgtb1-*2 ovules exhibit a WT-like localized signal (L) and a 40% sectorial signal in scattered cells of the outer integument and chalaza (O). (M) Graph summarizing DIIS-Venus signal localization in ovules at all analyzed stages of development. The white bar shows a typical signal at the chalaza and in the outer integument; examples in (A–D). The gray bar shows signal present only in scattered cells of the chalaza and outer integument; example in (O). The black bar shows no signal or a very weak signal; examples in (E–K, N). The exact number of ovules counted is given above each bar. Epifluorescence microscopy; merged images of DIC and the *35S:*DIIS-Venus signal. The white dotted line highlights the MMC, FM, or FG, as appropriate for the image. Abbreviations: ne, nucellar epidermis; ii, inner integument; oi, outer integument; f, funiculus; M, micropyle; CH, chalaza; ec, egg cell; sc, synergid cell; ccn, central cell nucleus; v, central vacuole. Scale bar=20 μm for all images. (This figure is available in color at *JXB* online.)

To complement the data obtained for the positive auxin reporter *pDR5rev*:3×Venus, we also utilized the DIIS-Venus negative reporter. In WT ovules, fluorescent signal was detected in the early stages of development, from the MMC to the first mitotic division (Fig. 7A–C), complementing the results obtained by *pDR5rev:*3×Venus. Around ovule maturity, DIIS-Venus signal was present in the outer integuments and the chalaza, and also more weakly in the outer cell files of the funiculus (Fig. 7D). No fluorescent signal was observed inside the embryo sac, as expected due to the *CaMV35S* promoter specificity that drives the DIIS-Venus expression.

The DIIS-Venus sensor signal was not detected in young *rgtb1* ovules (Fig. 7E–G, I–K versus A–C) and was hardly detected around maturity; <3% of ovules showed a WT-like DIIS-Venus signal at the FG6/7 stage (Fig. 7L). Overall, at maturity, 42% of *rgtb1* ovules had DIIS-Venus signal distributed unequally (sectorial) in single cells of the outer integument and chalaza (Fig. 7O). Moreover, the *rgtb1* ovules arrested at FM/FG2 never showed DIIS-Venus signal (Fig. 7H, N). The DIIS-Venus signal localization in *rgtb1* is summarized on a graph (Fig. 7M). The lack of DIIS-Venus signal in arrested ovules supported the *DR5rev:*3×Venus results where nucellar auxin accumulation was still maintained in young *rgtb1* ovules and those unable to complete megagametogenesis (compare Fig. 6).

It is important to note that in the case of both DIIS-Venus and *pDR5rev*:3×Venus reporters, only plants showing reporter fluorescence in other sporophytic tissues and at least some ovules were considered for observation and counting. Again, as was the case for PIN1–GFP and *DR5rev:*3×Venus, the ovules showing positive and negative signal for the DIIS-Venus reporter were distributed randomly in *rgtb1* ovaries.

### Multiple members of the RGT complex are expressed in ovules around meiosis

The results described above are consistent with *rgtb1* mutants, showing decreased efficiency of PIN1 protein recycling and auxin responses. This might be due to hypoprenylation of at least some Rab proteins in the *rgtb1* mutants, to the complete absence of RGT activity in ovules, or to a different Rab protein specificity of a remaining RGTB2 enzyme isoform at the FM stage. To address these possibilities, gene expression profiles were analyzed.

**Fig. 8.**
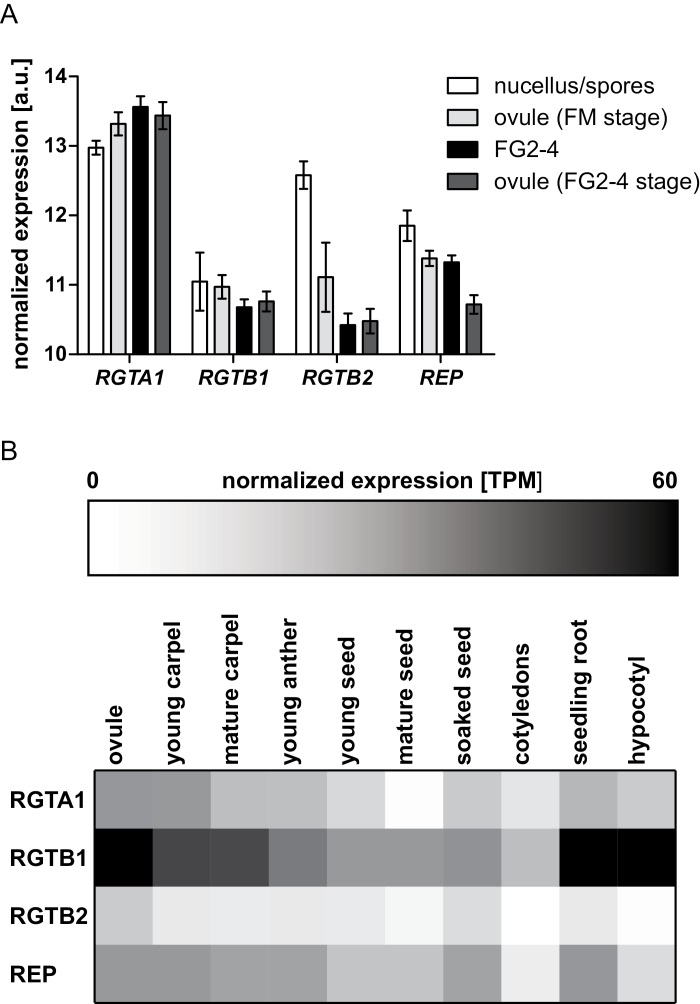
Expression profiles of RGT genes in ovules and other tissues. (A) Expression of RGT complex-encoding genes in WT ovules during megasporogenesis and development of 2–4 nucleate female gametophytes (FG2-4). Nucellar tissue and FG2-4 samples were laser microdissected and analyzed by hybridization to Affymetrix arrays as described in Tucker *et al.* (2012a). The rest of the ovule tissues from dissected samples were collected separately. Mean normalized gene expression values for the Col WT nucellus, female gametophyte, and whole ovule ±SD are presented. (B) RNASEQ reads from the experiment SRP075604 were downloaded from publicly available databases (Klepikova *et al.*, 2015, 2016). Reads were mapped to the Arabidopsis genome, and gene expression was calculated and presented as a heat map for selected plant organs.

The expression level of the *RGTA1*, *REP*, *RGTB1*, and *RGTB2* genes was examined in whole carpels, mature ovules, and seeds at different stages using published RNA-sequencing datasets (Klepikova *et al.*, 2015, 2016; Fig. 8). *RGTA1* and *REP* genes are expressed at relatively low levels in carpels, mature ovules, and seeds, as well as in all other studied tissues (Fig. 8B). *RGTB1* expression is generally uniform, but always higher than that of *RGTA1* and *REP*, while *RGTB2* expression is barely detectable. Expression datasets from young microdissected ovules were also analyzed, including nucellar cells at the time of meiosis/FM differentiation, FG2–4 female gametophytes, and whole ovules at the respective stages (Tucker *et al.*, 2012a). The data indicate that all members of the RGT complex are expressed in young ovules (Fig. 8A). The *RGTA1*, *RGTB2*, and *REP* genes show significant differences in expression between tissues; *RGTB2* and *REP* are up-regulated in nucellar cells relative to the whole ovule [fold change (FC)=2.8, *P*-value <0.01; and FC=1.4, *P*-value <0.05, respectively], while *RGTA1* is slightly more abundant in the whole ovule (FC=0.8, *P*<0.05) compared with its tip. *REP* is also up-regulated in the developing FG relative to the whole ovule (FC=1.5, *P*<0.05). Interestingly, in ovules around meiosis, the level of expression of *RGTB2* is similar to or higher than the levels of expression of *RGTB1* (Fig. 8A) in striking contrast to other tissues, where *RGTB2* transcript is always less abundant (Fig. 8B).

**Fig. 9.**
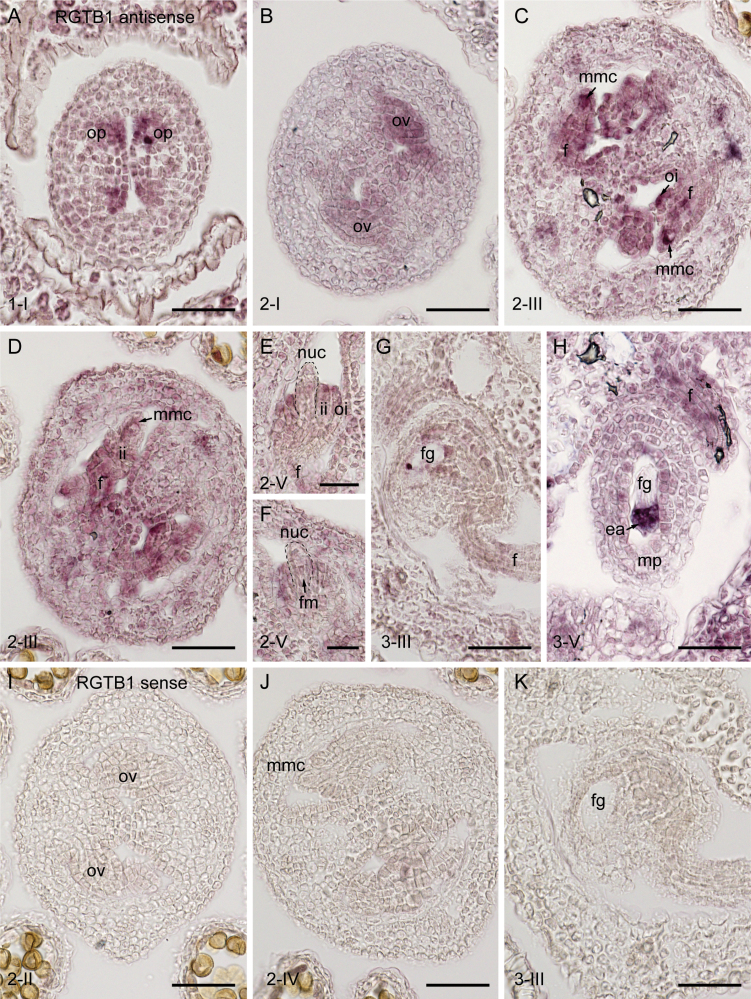
*In situ* hybridization of RGTB1 in ovule tissues. (A–H) Antisense *RGTB1* probe. (I–K) Sense *RGTB1* probe. During early stages of ovule development, *RGTB1* transcript was detected in a range of tissues including (A) ovule primordia, (B) all tissues of the young ovule, and (C) subsequently in the megaspore mother cell, chalaza, integuments, and funiculus. (D) During meiosis, signal diminished in the MMC but remained present in the chalaza, integuments, and funiculus. (E, F) During functional megaspore selection, *RGTB1* was expressed weakly or was absent in the nucellus/FM but strongly in the integuments. (G) Signal was detected in the female gametophyte during later stages of cell specification, and was abundant in the egg apparatus. Signal remained in sporophytic tissues such as the integuments and funiculus. (I–K) Sense probes showed no background hybridization. op, ovule primordia; mmc, megaspore mother cell; oi, outer integument; ii, inner integument; f, funiculus; nuc, nucellus; fg, female gametophyte; ea, egg apparatus; mp, micropyle; ov, ovule; fm, functional megaspore, Scale bar=50 µm in A–D and G–K, and 25 µm in E and F. (This figure is available in color at *JXB* online.)

To further delineate the spatial distribution of *RGTB1* mRNA in ovules, mRNA *in situ* hybridization was utilized. At early stages of ovule development, *RGTB1* mRNA was evenly distributed in the ovule primordia and ovule proper (Fig. 9A, B). During MMC expansion, *RGTB1* was detected throughout the ovule but was particularly strong in the archesporial cell/young MMC (Fig. 9C). In contrast, during meiosis, signal was strong in the developing integuments but was depleted in the nucellus and MMC (Fig. 9D). During subsequent stages, signal was not obvious in the developing FM or FG (Fig. 9E–G), but was detected in most sporophytic ovule tissues including the funiculus (Fig. 9E–G). As the ovule approached maturity, signal was maintained in the funiculus and was particularly abundant in the egg apparatus (Fig. 9H). These results indicate that RGTB1 mRNA partially overlaps with the sites of PIN accumulation, but is not abundant in the MMC or nucellus from meiosis until gametophyte cellularization.

### Rab family genes are expressed in young Arabidopsis ovules

Identification of the hypoprenylated Rab proteins in *rgtb1* mutants could provide further mechanistic information regarding the key Rab proteins required for ovule development. Unfortunately we were unable to find differences in Rab geranylgeranylation in WT versus *rgtb1* flowers by proteomic methods (Supplementary Table S1). In parallel, examination of the RNA sequencing (Supplementary Fig. S3B) and laser capture microdissection (Supplementary Fig. S3A) datasets suggests that multiple Rab-encoding genes are expressed in ovules at meiosis, during FG development, and at anthesis. All *RabD* genes are expressed, with *RabD2b* showing the most prominent expression level among all the Rabs at anthesis. In total, only 11 of the 55 Rab-encoding genes are not expressed in ovules. Notably, in post-meiotic ovules when the *rgtb1* mutant phenotype is first detected, the most abundant *Rab* transcripts are *RabE1d* and *RabH1b* followed by *RabB1c*; high expression of *RabA4a*, *RabA1b*, and *RabA5b* is also observed. Five *Rab* genes are significantly up-regulated in the nucellus compared with the rest of the ovule, namely *RabE1d*, *RabH1b*, *RabF2b*, *RabA1a*, and *RabA6a*. In contrast, 10 genes are more abundant in other parts of the ovule compared with the tip, and these include *RabA2a*, *RabD2b*, *RabA5a*, *RabA2b*, *RabA5c*, *RabD2c*, *RabG3c*, *RabG3d*, and *RabC2b*. Taken together, these results indicate that multiple *Rab* genes are expressed in the developing ovule at meiosis and these may be targets for hypoprenylation in *rgtb1*. The pattern of Rab gene expression changes during the time of ovule development and seems to differ in gametophytic versus maternal sporophytic tissues.

## Discussion

Earlier observations of *rgtb1* mutants suggested that many of their phenotypes may result from disturbed auxin homeostasis (Hala *et al.*, 2010). The most straightforward hypothesis linking defects in vesicular transport to a reduced auxin response is the disturbance of auxin carrier recycling, which potentially leads to defective formation of instructive auxin gradients in developing tissues and organs. Out of several auxin carriers, the efflux PIN proteins are well described to depend on vesicular traffic and basal/apical sorting (Luschnig and Vert, 2014). At least three PIN proteins are expressed in the developing ovule: PIN1, PIN3, and PIN6 (Larsson *et al.*, 2017). Of these three proteins, PIN1 is particularly important in early stages of ovule development, followed by PIN3 (Ceccato *et al.*, 2013). Others (including PIN6) take part in later events leading to correct formation of vascular bundles in the funiculus and auxin flux in and out of the mature FG and early embryo (Larsson *et al.*, 2017, Robert *et al.*, 2018). This prompted us to study the interplay of Rab-dependent vesicular traffic, PIN1, PIN3, and auxin at the early stages of ovule development, particularly during initiation of the female germline.

### Expression of *RGTB2* may contribute to the progression of meiosis and female megaspore differentiation in *rgtb1*

Transcriptomic analysis suggests that genes coding for *RGTA1* and *REP* subunits of the RGT complex are expressed at comparable levels in most plant tissues. The *RGTB* subunits are expressed in most organs in a ratio of ~1:10–1:20 *RGTB2* to *RGTB1*. The prominent exception is developing and mature pollen, where the expression of *RGTB2* equals or even exceeds that of *RGTB1* (Hala *et al.*, 2010). Here we show that RGTB1 mRNA is expressed in a range of ovule tissues including the young MMC, integuments, and funiculus. However, expression was weak or absent in the nucellus and FM. *In silico* evidence suggests that *RGTB2* is substantially expressed in the vicinity of the developing nucellus, FM, and FG, possibly even exceeding *RGTB1*. We speculate that additive tissue-specific expression of *RGTB1* and *RGTB2* in the ovule is required for normal germline development.

The hierarchy of Rab prenylation (Kohnke *et al.*, 2013) in Arabidopsis WT or *rgtb1* mutants was not addressed experimentally in previous studies (Hala *et al*., 2010; Gutkowska *et al.*, 2015), hence it is difficult to determine which particular Rab is responsible for the manifestation of *rgtb1* ovule phenotypes. The enzymatic activity of RGT in *rgtb1* is decreased to 25% of that of the WT, at least for some substrates such as Rab A2a (Hala *et al*., 2010). Biochemical studies, although not completely quantitative, may also indicate that the RGTB2 subunit has higher enzymatic activity for some Rab proteins than RGTB1 (Shi *et al.*, 2016). Hence, we also predict that there may be a compensatory effect of RGTB2 in *rgtb1* mutants, at least in the tissues or stages where *RGTB2* expression is relatively high, such as pollen and the young ovule. RGTB2 activity may facilitate completion of meiosis and FM specification in at least some *rgtb1* ovules, due to prenylation of important housekeeping Rabs. At the same time, other Rabs, being less abundant or having lower affinity for the RGTB2/RGTA1/REP complex, remain unprenylated and are easily degraded.

The timing of developmental arrest in *rgtb1* ovules is also interesting. Nucellus tissues isolated by laser capture microdissection at the meiosis/FM stage show high transcription of the *RGTB2* gene, while our *in situ* hybridization experiments suggest that *RGTB1* is weak or absent in the nucellus. Hence, RGTB2 may compensate for the lack of *RGTB1* transcript in the nucellus. Indeed both PIN1 and PIN3 localization and auxin accumulation during megasporogenesis were indistinguishable in the WT and *rgtb1*. At the FG2/FG4 stage, *RGTB2* transcript levels are low, while *RGTB1* is detected in the integuments, chalaza, and funiculus. At this developmental stage, FG arrest had already initiated in many *rgtb1* ovules, concurrent with PIN1 and PIN3 mislocalization from the basal membranes and an auxin concentration increase in the nucellus. This may suggest that the pool of Rabs prenylated (possibly by RGTB2) around meiosis was degraded and the number of Rab molecules still membrane bound and active is not sufficient to sustain the normal recycling of the endosomes. Meiosis, FM specification, and first mitotic division in Arabidopsis take >36 h (Schneitz *et al.*, 1995). Prenylated Rab protein levels on the cell membranes significantly drop after chemical inhibition of RGT after 48 h (Kazmierczak *et al.*, 2017), and the onset of acute systemic phenotypes in the absence of RGT activity was described to be at 4 d in vertebrate embryos (Moosajee *et al.*, 2009). This fits well with the timing of early FG development of Arabidopsis. Another possibility is that at the FM/FG2 stage, a Rab protein not expressed at earlier stages in the ovule is crucial for PIN1 recycling. Even if this Rab is synthesized correctly, the lack of geranylgeranyl modification renders it inactive, and PIN(s) recycling is compromised.

### Hypoprenylation of more than one Rab may explain the *rgtb1* ovule phenotype

In this work, we summarize data highlighting the expression of Rab-encoding genes in gametophytic and sporophytic tissues of the ovule. A number of Rab genes were up-regulated in nucellus tissue during megasporogenesis, at the same time that polarized PIN1 and PIN3 accumulate in nucellar epidermal cells. Interesting examples are members of the A and E families, known to perform cargo transport to the plasma membrane (Zheng *et al.*, 2005; Chow *et al.*, 2008; Camacho *et al.*, 2009; Asaoka *et al.*, 2013; Drdova *et al.*, 2013; Kirchhelle *et al.*, 2016). This gives a hint as to which Rabs may be involved in FG differentiation and development, although direct proof requires experimental confirmation.

Additional indications as to which Rab(s) may be involved in generative processes in *rgtb1* come from studies on individual/multiple Rab mutants. In particular, the *rabd2a/b/c* mutant is embryo lethal (Pinheiro *et al.*, 2009) and approximately half of the ovules in the *rabd2b/c* double mutant are not fertilized (Peng *et al.*, 2011). *Rab D2b* is the most abundant *Rab* transcript detected in the ovule at anthesis, and provided the highest score in the proteomic analysis of the flowers, indicating that RabD hypoprenylation in *rgtb1* may be a cause of some ovule phenotypes. Mutations in a common GEF for Arabidopsis RabF proteins, VPS9, causes embryo lethality, but no obvious seed deformations are detected (Goh *et al.*, 2007).

Neither RabD proteins nor RabF proteins have been associated with PIN1 and auxin localization, but the RabA subgroup may perform these functions. Studies highlight potential roles for RabA1 (Koh *et al.*, 2009; Feraru *et al.*, 2012; Naramoto *et al.*, 2014) or RabA5 (Drdova *et al.*, 2013) in this pathway, both of which are abundant in the young ovule. RabA1b and other members of the family take part in endosomal PIN1 protein recycling through VHA-a1-positive endosomes (Feraru *et al.*, 2012; Berson *et al.*, 2014). What is more, RabA1e-positive endosomes and PIN1 both show characteristic co-aggregation in protophloem cells in a sphingolipid biosynthesis mutant (Markham *et al.*, 2011). Interestingly, high VAN4 (RabA1 GEF) promoter activity is detected in procambium cells of leaves, shoots, and ovule funiculi (Naramoto *et al.*, 2014), marking the high demand for active RabA1 in vasculature-forming tissues. RabA1a and RabF2b expression was enriched in nucellus tissue during megasporogeneis.

### PIN1 and PIN3 internalization and incorrect auxin distribution in *rgtb1* ovules correlate with developmental arrest of the female gametophyte

The recycling of the main auxin efflux protein PIN1 and also PIN3 was affected in *rgtb1* ovules, leading to an aberrant *pDR5rev*:3×Venus expression, indicative of increased auxin concentration in sporophytic tissues surrounding the developing germline. In parallel, development of the germline was blocked in a significant fraction of *rgtb1* ovules, and these arrested ovules indeed showed unusually high and delocalized auxin maxima compared with adjoining ‘normal’ ovules. The symptoms of defective development in *rgtb1* ovules were primarily detected after the first mitotic division of the FM. Absence of the PIN1–GFP signal in the ovule provasculature was previously noted in another transport-related mutant, *hapless-13* (Wang *et al.*, 2016). FG arrest at the uni- to binuclear stage in *rgtb1* may be explained by a lack of PIN1 on the basal membranes of the provasculature cells in the chalazal part of the nucellus and in the funiculus, similar to that reported for the *pin1-5* mutant (Ceccato *et al.*, 2013).

Previous studies in Arabidopsis reported accumulation of auxin (via *pDR5rev* markers) in nucellus cell layers proximal to the FM (Pagnussat *et al.*, 2009), but not inside it. During subsequent stages of gametogenesis, auxin is not detected in the FG but reaches high concentrations in adjoining sporophytic cells. As FG development proceeds towards maturity, the auxin maximum diminishes and shifts from the micropylar to the chalazal pole of the ovule (Lituiev *et al.*, 2013). In many *rgtb1* ovules, *pDR5rev*:3×Venus signal remained strong at the micropylar pole at maturity, and this coincided with developmental arrest. It also coincided with mislocalized PIN1 and PIN3 in the funiculus, consistent with no correct path for auxin efflux. This implies that in WT plants auxin is produced in the young ovule in sporophytic tissues around the growing FG and exported out of the ovule to the mother plant through the funiculus, with the aid of PIN1 and PIN3 (Larsson *et al.*, 2017). This possibility is consistent with recent studies detailing analysis of auxin biosynthesis and transport in the Arabidopsis ovule (Panoli *et al.*, 2015; Larsson *et al.*, 2017; reviewed in Shirley *et al.*, 2019). In the case of *rgtb1*, the excess auxin in the nucellus may inhibit the progression of the FG towards maturity, which is consistent with the sporophytic nature of the germline abortion phenotype. Convincing complementary results were also obtained by the use of the DIIS auxin reporter. Alternatively, failed FG development inhibits progression of nucellar degradation, resulting in persistence of cells that accumulate auxin but are unable to transport it. These effects would have to be indirect and non-cell-autonomous, since we never observed auxin accumulation inside the FG.

### Small differences in auxin concentration in sister *rgtb1* ovules cause dramatic changes in fate

Interestingly, a diverse range of phenotypes were identified in *rgtb1* mutants, ranging from the complete cessation of FG development to formation of nearly normal mature ovules. Curiously, normal and affected ovules neighbor each other in one ovary; the situation where consecutive ovules are all affected or all normal is quite rare. We considered what the reason might be for unequal effects of the *rgtb1* mutation on sister ovules in the same ovary. PIN proteins are typically redundant in function, and the lack of one may induce expression of another (Vieten *et al.*, 2005).Furthermore, their expression (but not proper localization or recycling) is regulated by auxin presence in a positive feedback loop by AUX/IAA repressors, ARF and PLT transcription factors (reviewed in Habets and Offringa, 2014). If the auxin concentration is above a certain threshold, the hormone down-regulates PINs post-transcriptionally (Vieten *et al.*, 2005) or directly regulates the number of PIN proteins present at the membrane (Paciorek *et al.*, 2005; Robert *et al.*, 2010). This complex network of positive and negative feedback loops of auxin biosynthesis and transport enables fine-tuning of plant response to external and internal cues. The aberrant PIN1 localization (vesicles instead of basal membrane) in *rgtb1* mutants is consistent with decreased PIN recycling ability.

Deficiency of RGTB1 in *rgtb1* mutants leads to reduced Rab protein prenylation (Hala *et al.*, 2010). RGTB1 (or RGTB2 in the case of the *rgtb1* mutants) may contribute to tissue development in a dose-dependent manner, and that is why phenotypes appear occasionally in neighboring ovules. We hypothesize that in some ovules, the number of prenylated and membrane-attached Rabs is above the threshold to recycle the PIN1 efficiently to the basal membranes and pump auxin out of the ovule. In neighboring ovules, even slight changes in Rab abundance may not sustain enough recycling and the concentration of PIN on the membrane falls below this threshold. As a consequence, auxin becomes trapped in the nucellus, which leads to the observed persistence of auxin maxima around the FG, and inhibition of developmental progression. A similar case of inhibitory auxin action was previously reported for meristemoid cell differentiation into mature stomata mediated by PIN3 (Le *et al.*, 2014) and also in a *pin1-5* mutant, where the lack of PIN1 on basal membranes of the funiculus comes from lower production of this protein in the cells (Ceccato *et al.*, 2013). PIN and auxin regulatory networks are complex and the decisions on cell specification (e.g. to differentiate into a functional FG or stomata guard cell) appear to be made in any of the ovules/meristemoids independently. The reduced PIN1-mediated efflux in *rgtb1* mutants may prevent the establishment of gradients and/or transcriptional profiles that are required for the transition to gametogenesis.

### Sporophytic tissues of *rgtb1* ovules do not develop correctly

Defects in ovules of *rgtb1* mutants were not restricted to the nucellus and germline, since the integuments, which later give rise to seed coat, occasionally showed abnormal features. Similar phenotypes were previously reported in Arabidopsis ovules misexpressing auxin-dependent transcription factors (Wu *et al.*, 2006; Kelley *et al.*, 2012; Simonini *et al.*, 2016) or *Hieracium* ovules treated with the polar auxin transport inhibitor 1-*N*-naphthylphthalamic acid (NPA) (Tucker *et al.*, 2012b). Also the size and shape of *rgtb1* integument cells were often disturbed. Integuments with milder deformations apparently enabled normal growth of the FG.

To summarize, this study provides a comprehensive analysis of FG development in a vesicular transport-deficient plant, highlighting the key role of sporophytic vesicle transport in development of the haploid generation. Although further work is required to identify the particular Rab protein responsible for the observed *rgtb1* female gametogenesis-related phenotypes, we describe the influence of transport downturn on localization of PIN1 and PIN3 auxin efflux proteins and auxin gradient formation in the ovule. We suggest that this will encourage further, detailed studies on Rabs and involvement of their interactors in ovule development.

## Supplementary data

**Fig. S1.** Female sporogenesis in *rgtb1* mutants.

Fig. S2. Localization of PIN1–GFP, PIN3–GFP, and auxin sensors in *rgtb1* seedling roots.

Fig. S3. Expression of Rab-encoding genes in the ovule.

Table S1. Proteomic analysis of Rab proteins isolated from WT and *rgtb1* flowers.

The following supplementary data are available at *JXB* online.

eraa430_suppl_Supplementart_File001

## Data Availability

All data supporting the findings of this study are available within the paper and within its supplementary data published online.
