# Supplementary material for: Rab-dependent vesicular traffic affects female gametophyte development in Arabidopsis
Source: J Exp Bot. 2020 Sep 16;72(2):320–40. doi: 10.1093/jxb/eraa430 (PMC7853608; doi:10.1093/jxb/eraa430)
Supplement: eraa430_suppl_Supplementart_File001 [file eraa430_suppl_supplementart_file001.pdf]

MS ID#: JEXBOT/2020/284091

MS TITLE: Rab-dependent vesicular traffic affects female gametophyte development in Arabidopsis

Joanna Rojek [1], Matthew R. Tucker [2], Sara C. Pinto [2,7], Michał Rychłowski [3], Małgorzata Lichocka [4], Hana Soukupova [5], Julita Nowakowska [6], Jerzy Bohdanowicz [1], Gabriela Surmacz [4], Małgorzata Gutkowska [4, #]

[#] corresponding author

Małgorzata Gutkowska, gosiag@ibb.waw.pl,

Supporting information:

Figure S1. Female sporogenesis in *rgtb1* mutants.

Figure S2. PIN1-GFP, PIN3-GFP and auxin sensors localization in *rgtb1* seedling roots.

Figure S3. Expression of Rab encoding genes in the ovule.

Table S1. Proteomic analysis of Rab proteins isolated from WT and *rgtb1* flowers.

Availability statement: All data supporting the findings of this study are available

Figure S1. Female sporogenesis is similar in WT and *rgtb1* mutants.

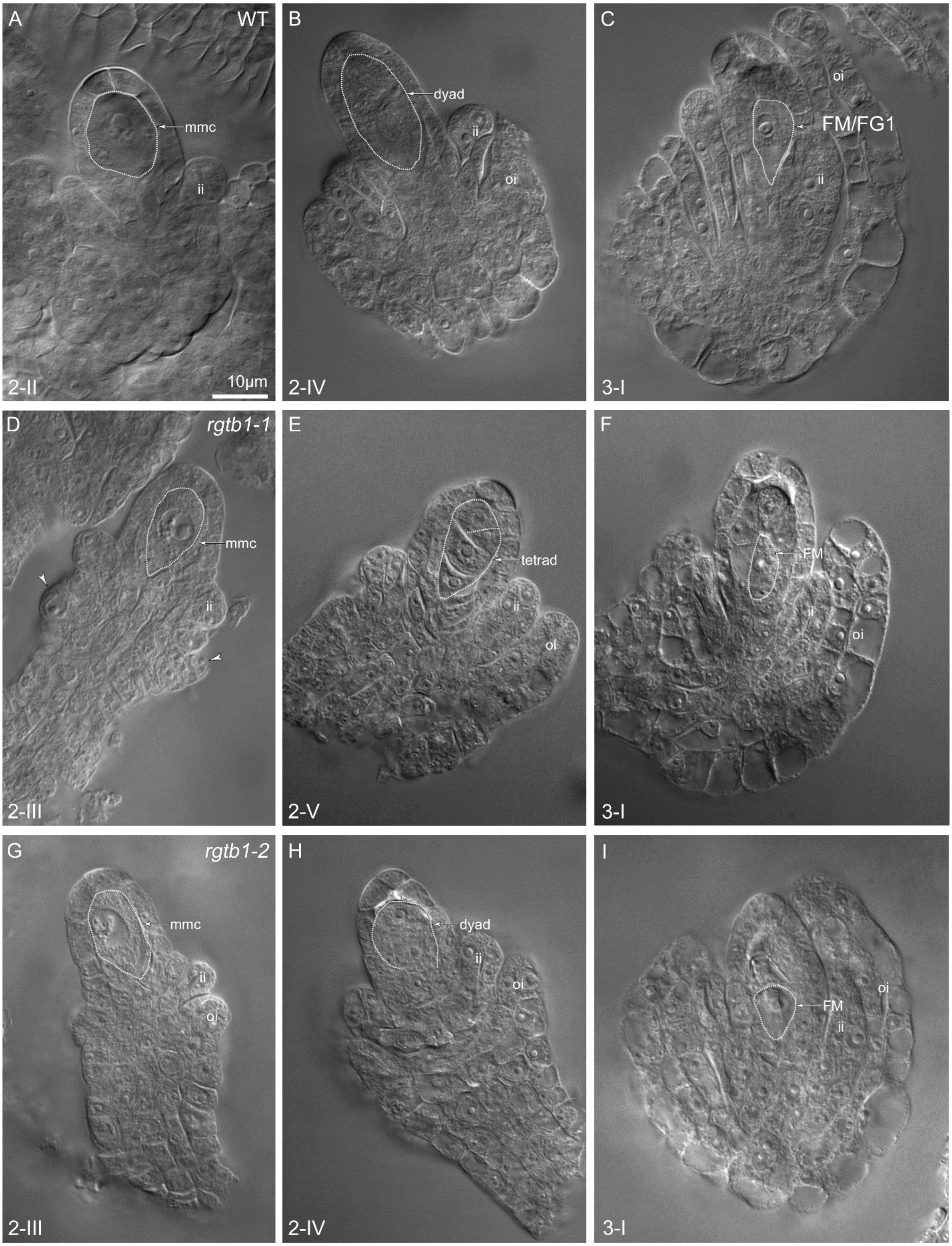

Female sporogenesis from stage 2-II to 3-I (according by Schneitz et al., 1995) in WT (A-C), *rgtb1-1* (D-F) and *rgtb1-2* (G-I). Despite morphological and cellular disruption of *rgtb1* ovules (e.g. F,I), sporogenesis proceeded from the MMC stage to the T-shape tetrad stage (H) and FM formation (F,I), similarly to sporogenesis in WT (A-C). DIC microscopy. Bar = 10 µm in (A) corresponds for all images. Method is described in a main text.

Figure S2. Detection of PIN1-GFP, PIN3-GFP and auxin sensors in WT and *rgtb1* seedling roots.

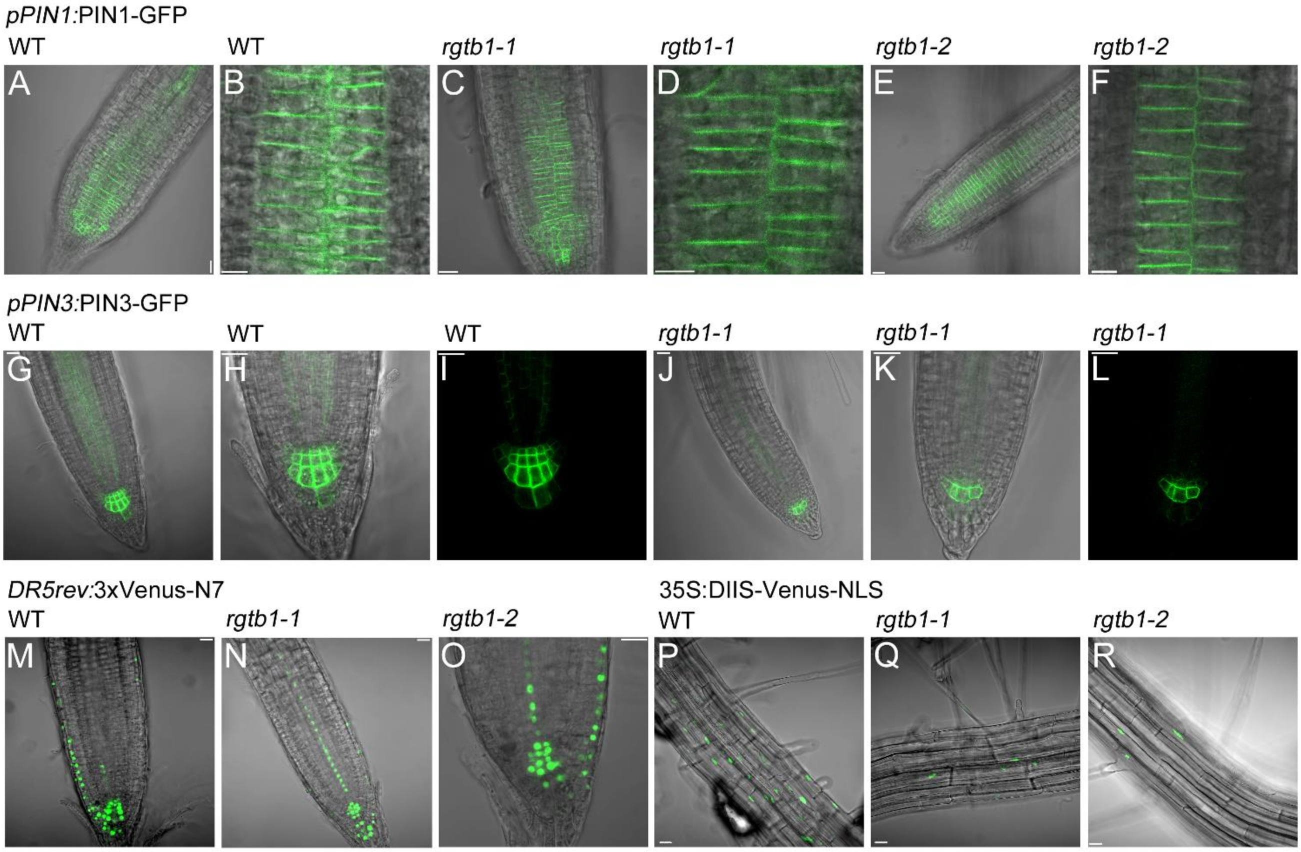

(A-F) *PIN1:PIN1-GFP* expression in roots. (G-L) *PIN3:PIN3-GFP* expression in roots. (M-O) *DR5rev:*3xVenus expression in roots. (P-R) DIIS-Venus signal in root epidermis. CLSM microscopy; merged images of DIC and fluorescence signal. Bar = 20 µm corresponds to A,C,E,G-N,P-Q images. Bar = 10 µm corresponds to B,D,F,O images.

Method:

Plants were grown on ½ MS vertical plates in a growth chamber (Percival, CLF Plant Climatics, Germany) under long day conditions. 10-day-old seedling roots were mounted in water and observed immediately under an Eclipse TE 2000E inverted confocal microscope (Nikon Instruments B.V. Europe, Amsterdam, The Netherlands) equipped with a 60x Plan-Apochromat oil immersion objective. GFP and Venus fusion proteins were excited with a Sapphire 488 nm laser (Coherent, Santa Clara, CA, USA) and observed using the 515/530 nm emission filter. Confocal images were analyzed using free viewer EZ-C1 software and Image J software.

Figure S3. Expression of Rab encoding genes in the ovule.

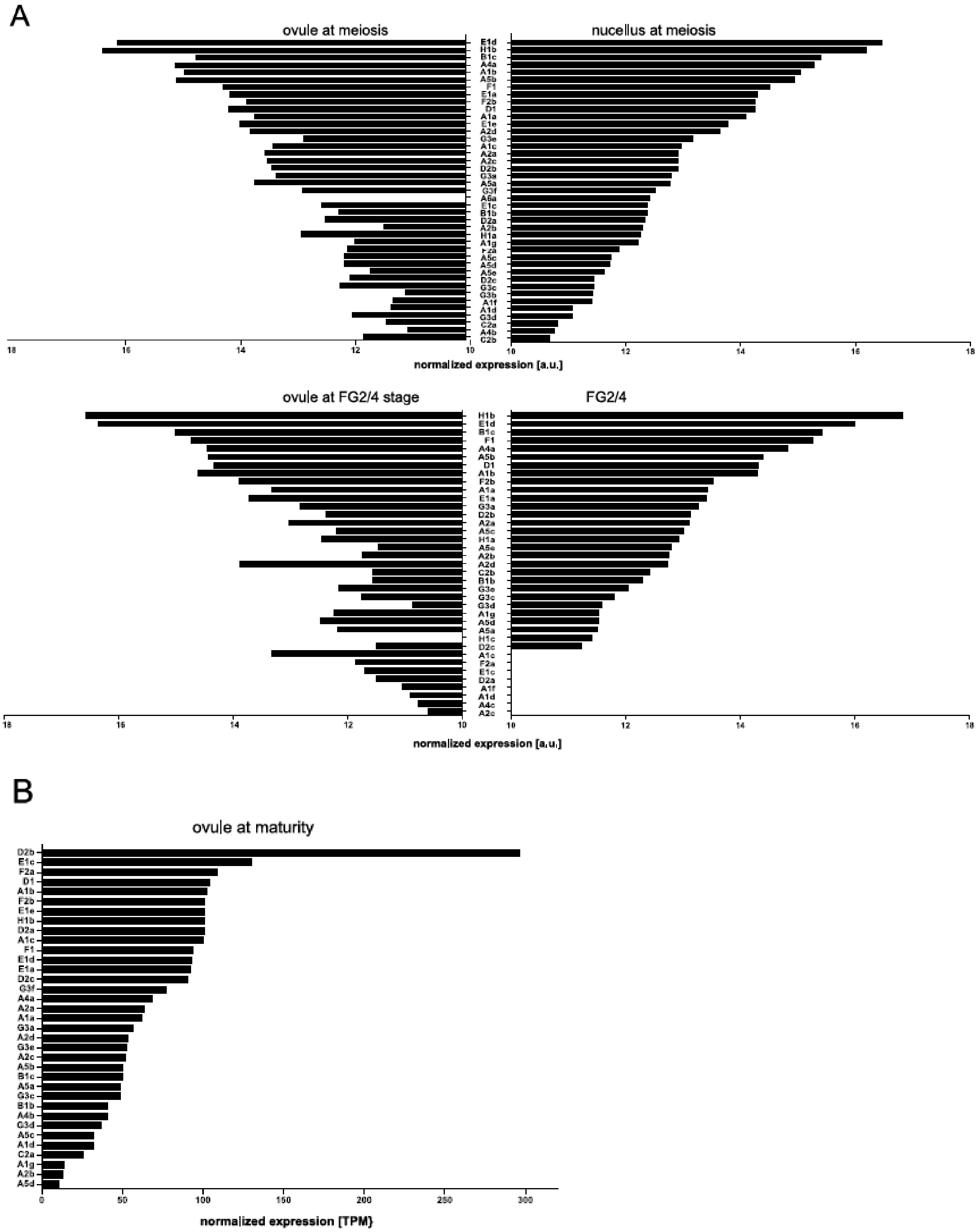

A) Expression of Rab encoding genes in WT ovules during megasporogenesis and development of 2-4 nucleate female gametophytes (FG2-4). Nucellar tissue and FG2-4 samples were laser microdissected and analysed (Tucker *et al*., 2012a). The rest of the ovule tissues from dissected samples were collected separately. Means of normalized gene expression values for the Col WT nucellus, female gametophyte and whole ovule +/- SD are presented. (B) RNASEQ reads from the experiment SRP075604 were downloaded from publically available databases (Klepikova *et al*., 2015; Klepikova *et al*., 2016).

Table S1. Proteomic analysis of Rab proteins isolated from WT and *rgtb1* flowers

**Protein name**	**Gene number**	**Mean peptide coverage +/- SD [%]**			**Number of peptides**
		**WT**	***rgtb1-1***	***rgtb1-2***	
Rab A1a,b,c,d,f,g	AT1G06400 AT1G16920 AT5G45750 AT4G18800 AT5G60860 AT3G15060	26.29 +/- 8.63	29.71 +/- 10.70	23.80 +/- 8.35	1-9
Rab A2a,b,c,d	AT1G09630 AT1G07410 AT3G46830 AT5G59150	29.33 +/- 13.26	35.17 +/- 10.98	22.8 +/- 9.88	2-10
Rab A4a,b,c,d	AT5G65270 AT4G39990 AT5G47960 AT3G12160	9.00 +/- 3.46	14.00 +/- 1.00	11.00 +/- 2.65	2-3
Rab A5a,b,c,e	AT5G47520 AT3G07410 AT2G43130 AT1G05810	10.50 +/- 4.95	16.00 +/- 4.24	7.00 +/- 3.46	1-5
Rab B1b,c	AT4G35860 AT4G17170	20.25 +/- 9.81	14.80 +/- 8.70	23.25 +/- 5.74	1-4
Rab C1	AT1G39950	17.67 +/- 3.05	19.33 +/- 5.13	10.67 +/- 4.51	1-6
Rab D1	AT3G11730	22.67 +/- 9.81	29.50 +/- 14.85	31.67 +/- 3.79	3-8
Rab D2a	AT1G02130	24.00 +/- 0.00	19.67 +/- 3.79	24.00 +/- 5.66	3-5
Rab D2b,c	AT5G47200 AT4G17530	55.67 +/- 5.77	55.00 +/- 4.53	54.80 +/- 2.49	9-15
Rab E1a,c,d	AT3G53610 AT3G46060 AT5G03520	28.33 +/- 7.63	30.29 +/- 10.16	34.57 +/- 8.42	4-9
Rab F1	AT3G54840	n.a. (10.00)	12.5 +/- 3.53	9.00 +/- 1.73	1-5
Rab F2a,b	AT5G45130 AT4G19640	n.a. (12.00)	19.00 +/- 7.94	24.67 +/- 5.68	1-4
Rab G3a,b,c,d,e,f	AT4G09720 AT1G22740 AT3G16100 AT1G52280 AT1G49300 AT3G18820	24.00 +/- 13.19	26.00 +/- 7.91	22.44 +/- 6.08	2-10
Rab H1b,d	AT2G44610 AT2G22290	18.00 +/- 11.31	27.00 +/- 1.41	23.00 +/- 2.83	3-8

Lysates from flowers from 6-weeks-old WT and *rgtb1* plants were resolved on SDS-PAGE gels and bands corresponding to mass 17-30 kDa were cut, trypsinized and analysed by LC-MS/MS. Data come from three independent plant cultivations, each containing at least five plants. Mean peptide coverage +/- SD is shown for each identified protein. Only proteins present in all three experiments were taken into account.

To confirm the transcription data we performed MS analysis in order to detect and compare Rab protein prenylation in WT and *rgtb1* flowers. The flower proteome of *Arabidopsis* mirrored well the more spatially and temporarily resolved transcriptome. However, due to the absence of positively ionized amino acid residues close to the C-terminus of any of the Rab proteins (and hence a lack of ionized peptides containing prenylatable cysteines) we were unable to find any difference in peptide geranylgeranylation in *rgtb1* versus WT.

Method:

Flower buds and open flowers from WT and *rgtb1* plants were collected, snap frozen in liquid nitrogen and ground. Homogenates were centrifuged and supernatants were boiled in Laemmlie buffer and resolved on SDS-PAGE. Protein bands corresponding to the 17-30 kDa region were cut from the gel, digested with trypsin and analyzed by liquid chromatography coupled to a LTQ FT ICR mass spectrometer (Hybrid-2D-Linear Quadrupole Ion Trap – Fourier Transform Ion Cyclotron Resonance Mass Spectrometer, Thermo Electron Corp., San Jose, CA). Acquired raw data were processed by Mascot Distiller followed by Mascot Search (Matrix Science, London, UK) against the TAIR database allowing for geranylgeranylation modification. MS analysis was performed at Mass Spectrometry Laboratory, IBB PAS. Mean peptide coverage and SD were calculated from three repeated independent analyses for a given genotype.
